# Prevalence of lifetime self-injurious thoughts and behaviors in a global sample of 599 patients reporting prospectively confirmed diagnosis with premenstrual dysphoric disorder

**DOI:** 10.1186/s12888-022-03851-0

**Published:** 2022-03-19

**Authors:** Tory Eisenlohr-Moul, Madeline Divine, Katja Schmalenberger, Laura Murphy, Brett Buchert, Melissa Wagner-Schuman, Alyssa Kania, Sabina Raja, Adam Bryant Miller, Jordan Barone, Jaclyn Ross

**Affiliations:** 1 Department of Psychiatry, University of Illinois at Chicago, 912 South Wood Street, Chicago, IL 60612 USA; 2 International Association for Premenstrual Disorders, Boston, MA USA; 3 University of Texas at Austin, Austin, TX USA; 4 Heidelberg University, Heidelberg, Germany; 5 University of Cincinnati, Cincinnati, OH USA; 6 University of North Carolina at Chapel Hill, Chapel Hill, NC USA; 7 RTI International, Raleigh, NC USA

**Keywords:** Premenstrual dysphoric disorder, Premenstrual syndrome, Menstrual cycle, Hormones, Suicide

## Abstract

**Background:**

Suicide is the second leading cause of death among Americans ages 10 to 34, with alarming recent increases in suicide rates among those assigned female at birth. A large body of evidence points to menstrual cycle influences on self-injurious thoughts and behaviors (STBs), suggesting that neurobiological hormone sensitivities, such as in premenstrual dysphoric disorder (PMDD), may drive suicide risk in females. However, existing studies of STBs in PMDD use cross-sectional self-report measures of PMDD with poor validity. As a first step to establish accurate prevalence rates of STBs in PMDD, we examined the lifetime prevalence of STBs in a large global survey of patients reporting a diagnosis of PMDD based on daily ratings.

**Method:**

Individuals with self-reported PMDD symptoms were invited to an online survey through online support groups for PMDD and social media posts from PMDD awareness accounts. Participants reported demographics, whether they had been diagnosed with PMDD by a healthcare provider using daily ratings, STBs using the Columbia Suicide Severity Rating Scale, and history of lifetime comorbid psychiatric diagnoses.

**Results:**

Of 2,689 survey completers, 599 (23%) reported a diagnosis with PMDD based on two months of daily ratings and were included in analyses. We observed high rates of lifetime active suicidal ideation (72%), planning (49%), intent (42%), preparing for an attempt (40%), and attempt (34%), as well as non-suicidal self-injury (51%). The majority (70%) of the sample reported at least one lifetime comorbid psychiatric diagnosis. Predictors of lifetime active suicidal ideation included nulliparity, low-to-moderate (vs. high) income, and history of diagnosis with major depression or post-traumatic stress disorder. Predictors of lifetime attempts among those reporting lifetime active ideation included older age, nulliparity, lower income, and history of diagnosis with post-traumatic stress disorder or borderline personality disorder.

**Conclusions:**

These data indicate high rates of STBs among those reporting prospective diagnosis of PMDD and highlight the need for prospective research on mechanisms and prevention of STBs in PMDD. Clinical practice guidelines for PMDD should accommodate comorbidities and recommend frequent screenings for STB risk. STBs should be considered for inclusion in future iterations of the DSM PMDD diagnostic criteria.

**Supplementary Information:**

The online version contains supplementary material available at 10.1186/s12888-022-03851-0.

## Introduction

### Self-injurious thoughts and behaviors (STBs) and their association with ovarian hormone change

Self-injurious thoughts and behaviors (STBs) represent a growing public health crisis: suicide is the second leading cause of death among Americans aged 10 to 34, and the fourth leading cause of death among Americans aged 35 to 54 [1]. Recent reviews highlight the need to identify factors that (1) predict time-varying changes in imminent risk for suicide and (2) predict the likelihood of suicide attempt among those reporting suicidal ideation (SI [2, 3]). Although those assigned male at birth die by suicide more frequently than those assigned female at birth (AFAB), AFAB are three times more likely to report suicidal thoughts, one and a half times more likely to report non-suicidal self-injury [4], and three times more likely to report a suicide attempt [5, 6]. Although the etiology of these sex differences in STBs likely involves various sociocultural factors, ovarian hormone fluctuations may also play an important role in both short- and long-term STB risk. In support of this notion, the sex difference in STBs manifests between puberty [7] and menopause [8], when significant ovarian hormone fluctuations are present [9]. Most notably, accumulating cross-sectional studies indicate that suicide attempts and deaths are most likely to occur during the premenstrual and menstrual weeks of the menstrual cycle compared to other phases [10, 11].

### Inter-individual differences in hormone sensitivity and PMDD

The effects of the menstrual cycle on behavior have been the subject of psychological research for decades, and daily symptom tracking studies (i.e., prospective studies*)* find that these changes are abnormal, representing a pathological response in a subset of hormone-sensitive individuals. Roughly 5.5% of the naturally-cycling population appears to suffer from these cyclical emotional and somatic symptoms, which typically emerge in the luteal phase of the menstrual cycle (i.e., two weeks before menses) and remit during or after menses [12]. Experimental studies revealed that these premenstrual symptoms, consistent with the new DSM-5 diagnosis of premenstrual dysphoric disorder (PMDD; [13]), are caused by an abnormal neurobiological sensitivity to normal changes in ovarian hormones and their metabolites across the menstrual cycle [14, 15]. Therefore, the increased risk of STBs observed in reproductive-age AFAB may be in part driven by individuals with PMDD, who are neurobiologically sensitive to the natural hormone changes associated with the menstrual cycle.

### Major limitations of retrospective report in PMDD research

Many patients who report PMDD symptoms in cross-sectional surveys or interviews (i.e., retrospective report) fail to show a pattern of cyclical change when symptoms are tracked daily across the cycle, which renders currently-available cross-sectional symptom measures invalid for determining the presence of premenstrual symptoms or PMDD [16–18]. This has led to the DSM-5 PMDD requirement that diagnosis be confirmed by daily symptom charting across two menstrual cycles [19].

Although a systematic review of existing literature on STBs and premenstrual symptoms highlighted a consistently positive association between self-reported premenstrual symptoms and STB risk [20], these data fail to provide convincing evidence of an association because each individual study utilized retrospective (cross-sectional) measures to diagnose PMDD, which do not outperform chance in identifying actual cases of PMDD [16–18, 21]. Therefore, there is a need to determine the prevalence of STBs among individuals who have been prospectively diagnosed with PMDD.

### The role of psychiatric comorbidities in STBs among those diagnosed with PMDD

The role of comorbidities in shaping the impact of PMDD on STBs and other important clinical outcomes remains largely unstudied and needs to be evaluated more thoroughly. While the DSM-5 requires at least 5 symptoms of PMDD to be fully confined to the luteal phase rather than merely the exacerbation of chronic disorder, the presence of comorbid psychiatric disorders (wherein symptoms are substantively different from those observed cyclically and are present throughout the cycle) may be the rule rather than the exception among those with PMDD [22]. Nonetheless, most published studies of prospectively-confirmed PMDD exclude those with a comorbid psychiatric diagnoses, presumably to increase confidence in the cyclical nature of symptoms and to increase sensitivity to detect cyclical mechanisms. However, this ubiquitous research practice may give clinicians the inaccurate impression that comorbidities are exclusionary for a DSM-5 diagnosis. Relatedly, since STBs are not included in the diagnostic criteria for PMDD, clinicians may assume that increased STB risk in PMDD is attributable to psychiatric comorbidities, such as comorbid mood disorders, rather than PMDD itself. Therefore, there is a need to investigate the possible impact of psychiatric comorbidities on outcomes in PMDD. As a first step, the present study examines the associations of various psychiatric disorders with STBs (lifetime SI, lifetime suicide attempt among those with lifetime SI) in patients reporting prospectively diagnosed PMDD.

### Current study and hypotheses

We utilized data from the 2018 Global Survey of Premenstrual Disorders (2018 GSPD), a patient-centered research initiative performed by a collective of nonprofit organizations focused on premenstrual disorders (International Association for Premenstrual Disorders/IAPMD, Vicious Cycle, and Me v PMDD Symptom Tracker).

The first goal of the present study was to describe the lifetime prevalence of STBs in a global sample of AFAB people reporting a prospective (i.e., using daily ratings) diagnosis of PMDD by a healthcare provider. Moreover, we aimed to clarify whether the lifetime prevalence of STBs depended upon the presence of self-reported lifetime psychiatric comorbidity. We hypothesize that prospectively confirmed PMDD is characterized by higher absolute lifetime prevalence rates for SI and attempts than the general population, and that this will hold true regardless of whether a lifetime diagnosis of comorbid psychiatric disorder is reported. Further, we hypothesize that those with PMDD and a psychiatric comorbidity will show significantly higher prevalence of all STBs than those with PMDD only.

The second goal was to determine the association of demographics and specific self-reported comorbid psychiatric diagnoses with the likelihood of lifetime active SI and the likelihood of lifetime suicide attempt among those with lifetime active SI.

## Materials and methods

### Study design

All methods were carried out in accordance with the Declaration of Helsinki. Individuals were recruited through social media accounts belonging to organizations focused on PMDD awareness, peer support, education, and advocacy. Recruitment posts and emails read: "Tell us about your experience of PMDD," and "Help others with PMDD by reporting about what it's like for you." Interested parties were directed to an online survey that measured demographics, nationality, diagnostic status, treatment history, and impact of symptoms across areas of their life. At the start of the survey, all content areas were presented, participants were told that all survey responses were optional and anonymous, and participants provided informed consent. Participants were presented with an additional consent page prior to questions about suicide that explained the nature of the upcoming items and were given the option to skip the section. Participants were provided no compensation for completing the survey. Data collection was carried out between September 24, 2018 to December 4, 2018. Duplicate surveys submitted from the same IP address were deleted. Following the completion of data collection and duplicate deletion, data were de-identified and shared with the first author, who obtained approval for the analytic project from the University of Illinois at Chicago Institutional Review Board.

### Measures

#### Self-report of confirmed PMDD diagnosis using daily ratings

The primary goal of this archival analysis project was to characterize lifetime STB risk in those reporting a diagnosis of PMDD by a medical provider using daily ratings; therefore, the sample was restricted to only those reporting a prospectively confirmed diagnosis of PMDD by a medical provider. For this purpose, we asked, "Has a medical professional ever examined daily symptom ratings across two cycles to determine a diagnosis of PMDD?". Participants responded, ‘‘Yes, and they diagnosed me with PMDD’’, ‘‘Yes, and they diagnosed me with premenstrual exacerbation of an underlying disorder (PME)”, ‘‘Yes, and they diagnosed me with another, noncyclical disorder (not PMDD or PME)’’, or ‘‘No’’. Only those who selected the first item were included in the sample for the present study. While this criterion almost certainly reduces our sensitivity to detect true PMDD cases in the sample (e.g., excluding those who were never asked to provide daily ratings, or those whose providers failed to detect true PMDD patterns in the daily ratings), we chose the criterion to improve specificity relative to cross-sectional self-report, overcoming the limited validity of premenstrual measures used in prior work in this area.

#### Self-reported lifetime diagnosis with other psychiatric disorders

A secondary goal of our paper was to examine whether self-reported histories of various psychiatric diagnoses impact lifetime STB risk among those with PMDD, and which co-occurring diagnoses might be most strongly associated with STBs. For this purpose, we utilized the question, "Has a health care provider ever diagnosed you with any of the following conditions *in addition to* PMDD?", after which participants could endorse as many options as relevant. Psychiatric conditions listed included major depressive disorder (MDD), bipolar disorder, post-traumatic stress disorder (PTSD), any anxiety disorder, attention deficit hyperactivity disorder (ADHD), substance use disorder (SUD), eating disorder (ED), borderline personality disorder (borderline PD), and autism-spectrum disorder (ASD). Each was coded as present (1) or absent (0). Participants were later asked in an open-ended manner whether they felt that any of their psychiatric diagnoses had been misdiagnosed and were better explained by PMDD. While this was relatively rare (*n* = 12), disorders listed in this section were recoded as absent.

#### Lifetime suicidal ideation and behavior

Lifetime STBs were assessed using an abbreviated, online version of the Columbia-Suicide Severity Rating Scale (C-SSRS) [23]. C-SSRS is an extensively validated, semi-structured interview widely used in clinical and research settings due to its multi-dimensional approach to delineating suicidal ideation (SI), behavior, and intent. Based on the original C-SSRS, we adapted a self-report questionnaire to evaluate passive SI ("Have you ever wished that you were dead or wished that you could go to sleep and not wake up?"), active SI ("Have you ever actually had thoughts of killing yourself?"), suicidal planning ("Have you ever thought about how you might kill yourself?"), suicidal intent ("Have you ever had a suicide plan that you had some intention of acting on?"), suicidal preparation ("Have you ever taken any steps towards making a suicide attempt or preparing to kill yourself—such as collecting pills, getting a gun, giving valuables away or writing a suicide note?"), and actual suicide attempt ("Have you ever made a suicide attempt in which you carried out your plans?"). Participants were also asked about non-suicidal self-injury ("Have you ever performed self-harm behaviors, without an intention to kill yourself?") and history of hospitalization in the premenstrual phase ("Have you ever been admitted to a hospital or crisis care setting during the premenstrual phase?"). All outcomes were coded as yes (1) or no (0).

#### Analytic plan

Analyses were carried out in jamovi. First, we excluded those participants who declined to answer STB-related questions, as well as those who did not report a prospectively confirmed healthcare diagnosis with PMDD. Before moving on to our primary analyses, we used mean comparison techniques to test for demographic and STB differences between those who were excluded from the present sample and our primary study sample (those with prospectively confirmed PMDD); the purpose of this step was to describe any significant differences between the larger sample of those who had received a prospective diagnosis. For our primary analyses, we first calculated descriptive statistics to examine the prevalence of various STBs. This process was repeated for two subsamples – those who either did or did not report lifetime diagnosis with at least one psychiatric comorbidity—after which we used appropriate mean comparison techniques to determine whether the two groups significantly differed on each variable. Next, we used logistic regression models predicting the lifetime presence of active SI to examine which demographics and self-reported psychiatric diagnoses were associated with risk. Finally, in the subsample of individuals with PMDD reporting lifetime active SI, we created a logistic regression model predicting the presence of suicidal behavior from the presence of all comorbid diagnoses, controlling for demographics (i.e., predicting lifetime attempt among those with lifetime active SI). Post-hoc power analyses were not carried out; however, sensitivity analysis dictates that the smallest detectible chi square value for a dichotomous predictor of a dichotomous outcome (e.g., critical value for association of comorbidity with ideation) is around 3.84 (*df* = 1, *α* = 0.05, 1-*β* = 0.80).

## Results

### Participant flow

Two thousand six hundred eighty-nine unique participants completed the survey. Of those, 2,542 (94.5%) consented to answer questions about their lifetime histories of STBs, and these respondents form the sample for the present study. Of those who provided STB data, 599 (23%) reported a provider diagnosis with PMDD based on two months of daily ratings.

#### Comparing those with and without a prospective diagnosis on demographic and study variables

Those with (vs. those without) a prospective diagnosis of PMDD did not differ on any measured demographic variable, although those with prospectively confirmed PMDD were significantly more likely to report any psychiatric comorbidity (*χ*^2^ (1) = 9.06, *p* = 0.0026), active SI (*χ*^2^ (1) = 8.84, *p* = 0.012), planning (*χ*^2^ (1) = 9.12, *p* = 0.010), intent (*χ*^2^ (1) = 9.88, *p* = 0.0072), and attempt (*χ*^2^ (1) = 5.30, *p* = 0.021). In addition, those with a prospectively-confirmed diagnosis of PMDD were more likely to report a history of psychiatric hospitalization for STBs in the premenstrual phase (*χ*^2^ (1) = 53.23, *p* =  < 0.0001). Therefore, the subsample with prospectively confirmed PMDD reported significantly more STBs than those without prospectively confirmed PMDD.

#### Descriptives in the prospectively diagnosed sample and by psychiatric comorbidity status

The first column of Table 1 presents descriptive statistics for all study variables in the prospectively diagnosed sample (*N* = 599). Most of the sample (*N* = 420; 70%) reported that they had received at least one comorbid psychiatric diagnosis (that they did not identify to be a misdiagnosis of their PMDD). All respondents reported being AFAB, and 97.8% of the sample identified as cisgender. The age range was broad (13–62), although most of the sample were currently of typical reproductive age. About 93% of the sample reported Caucasian race and non-Hispanic ethnicity. Roughly a quarter of the sample reported a minority (non-heterosexual) sexual orientation. Participants from English-speaking countries dominated the sample (92.4%), including those from the United States (*N* = 296; 49.41%), the United Kingdom of Great Britain & Northern Ireland (*N* = 141; 23.53%), Australia (*N* = 58, 9.68%), Canada (*N* = 46, 7.67%), Ireland (*N* = 13, 2.17%), and Sweden (*N* = 11; 1.83%), with the remaining participants hailing from Mexico, The Netherlands, New Zealand, South Africa, Columbia, Costa Rica, Denmark, Ecuador, El Salvador, Finland, France, Guatemala, Indonesia, Norway, the Philippines, Romania, Saudi Arabia, Spain, Switzerland, Trinidad and Tobago, Uruguay, and Botswana (each < 10 participants). Participants' level of education and family income varied widely, with a bachelor's degree and $50,000–79,000 being the modal education and income levels, respectively. Roughly half of the sample reported having given birth to at least one biological child. Zero-order correlations (in the prospectively diagnosed sample) among all study variables are presented in Supplemental Table 1.

**Table 1:** Demographics in the full Prospectively diagnosed sample and by self-reported psychiatric comorbidity status

	**Full Prospectively Diagnosed PMDD Sample** **(*N* = 599)**		**PMDD with Hx of Psychiatric Comorbidity** **(*N* = 420)**		**PMDD without Hx of Psychiatric Comorbidity** **(*N* = 179)**		**Comparison**
	N	%	N	%	N	%	
**Age***, M (SD)*	34.23	7.87	33.91	8.17	34.36	7.74	*t*(597) = -.64, *p* = .52 *Cohen’s d* = -.057
**Gender**							*χ*^2^ (2) = 3.15, *p* = .20 Cramer’s V = .073
Female	586	97.830	408	97.143	178	178	
Non-binary	12	2.00	11	2.619	1	1	
Other	1	0.16	1	0.238	0	0	
**Gender Minority**	13	2.17	12	2.85	1	.56	*χ*^2^ (1) = 3.12, *p* = .07 Cramer’s V = .072
**Sexual Orientation**							*χ*^2^ (5) = 19.94, *p* = .001 Cramer’s V = .18
Straight	450	75.125	295	70.238	155	86.592	
Asexual	5	0.835	5	1.190	0	0.000	
Bisexual	74	12.354	61	14.524	13	7.263	
Gay/Lesbian	10	1.669	9	2.143	1	0.559	
Other	58	9.683	49	11.667	9	5.028	
Questioning/Unsure	2	0.334	1	0.238	1	0.559	
**Sexual Orientation Minority**	149	24.88	125	29.76	24	13.40	*χ*^2^ (1) = 17.96, *p* < .001 Cramer’s V = .17
**Race**							*χ*^2^ (3) = 8.59, p = .03 Cramer’s V = .12
White/Caucasian	551	91.987	381	90.714	170	94.972	
Asian or Pacific Islander	10	1.669	5	1.190	5	2.793	
Black or African	9	1.503	8	1.905	1	0.559	
American Indian or Alaskan Native	0	0.00	0	0.00	0.00	0.00	
Other	28	4.674	25	5.952	3	1.676	
Missing	1	0.167	1	0.238	0	0.000	
**Ethnicity**							*χ*^2^ (1) = 1.27, p = .25 Cramer’s V = .05
Hispanic or Latinx	25	4.17	15	3.57	10	5.58	
**Education**							*χ*^2^ (10) = 9.35, *p* = .49 Cramer’s V = .12
Bachelor's Degree	218	36.394	152	36.190	66	36.872	
Apprenticeship or Technical/Vocational Training	19	3.172	9	2.143	10	5.587	
Associate's Degree, HND, HNC, CEGEP, Higher Ed Certification	79	13.189	57	13.571	22	12.291	
In MS or HS	9	1.503	5	1.190	4	2.235	
Interrupted HS	17	2.838	11	2.619	6	3.352	
Completed HS/GED	111	18.531	77	18.333	34	18.994	
Interrupted College (no degree, not in school)	12	2.003	8	1.905	4	2.235	
In College	11	1.836	10	2.381	1	0.559	
In Grad School	5	0.835	4	0.952	1	0.559	
Masters Degree	106	17.696	78	18.571	28	15.642	
Doctoral Degree (PhD,MD,JD,DVM)	12	2.003	9	2.143	3	1.676	
**Income**							*χ*^2^ (10) = 5.43, *p* = .86 Cramer’s V = .10
$50,000—$79,999 USD	139	23.205	101	24.048	38	21.229	
less than $15,000 USD	94	15.693	67	15.952	27	15.084	
Student with No Income	9	1.503	6	1.429	3	1.676	
$15,000—$19,999 USD	33	5.509	25	5.952	8	4.469	
$20,000—$24,999 USD	29	4.841	16	3.810	13	7.263	
$25,000—$29,999 USD	26	4.341	18	4.286	8	4.469	
$30,000—$34,999 USD	29	4.841	18	4.286	11	6.145	
$35,000—$39,999 USD	35	5.843	24	5.714	11	6.145	
$40,000—$49,999 USD	64	10.684	46	10.952	18	10.056	
$80,000—$99,999 USD	46	7.679	31	7.381	15	8.380	
$100,000 USD or above	95	15.860	68	16.190	27	15.084	
**Parity** (any biological children)	278	46.41	218	51.90	60	33.52	*χ*^2^ (1) = 17.05, *p* < .001 Cramer’s V = .17
**Age at Menarche**	12.38	1.58	12.37	1.61	12.41	1.52	*t*(595) = 0.25, *p* = 0.80 *Cohen’s d* = .022

A few significant demographic differences (see Table 1) emerged between those who did versus did not report at least one psychiatric comorbidity. Those with a psychiatric comorbidity were less likely to be heterosexual or white, consistent with evidence that minoritized identities are associated with increased psychiatric risk due to minority stress– that is, due to the impacts of persistent global racism [24] and homophobia [25]. In addition, although those reporting psychiatric comorbidities did not differ in age from those who did not report a comorbidity, they were more likely to report having given birth to at least one child.

### Prevalence of STBs in the full prospectively diagnosed sample

**Fig. 1:**
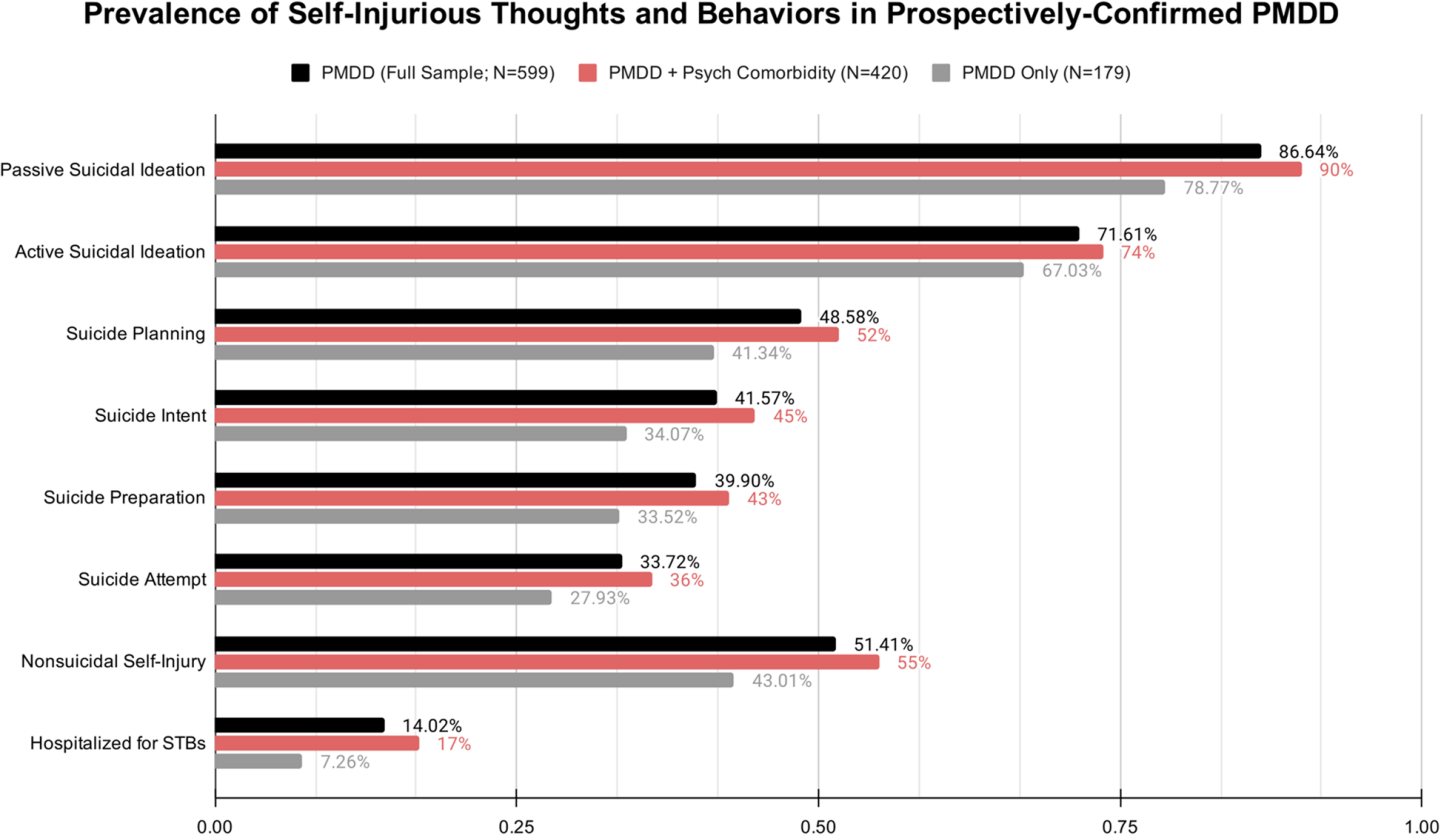
Prevalence of self-injurious thoughts and behaviors in those with prospectively confirmed PMDD, subdivided by psychiatry comorbidity status

The lifetime prevalence of STBs in the full prospectively diagnosed sample is presented in the first column of Table 2 and is represented by the black bars in Fig. 1. In the full prospectively diagnosed sample (*N* = 599), over eighty percent reported a lifetime history of passive SI, roughly three quarters reported a lifetime history of active SI, roughly half reported a history of suicidal planning, with two fifths reporting intent and preparation, and about one third reporting a history of attempt. Half the sample reported a history of non-suicidal self-injury. Roughly 14 percent reported a history of hospitalization for STBs around the premenstrual phase, although this finding should be interpreted with caution as we do not have access to confirmed cycle phase timing at hospitalization. Consistent with our predictions, these rates are well above cross-national population averages for lifetime prevalence of STBs, including active SI (9.2% in general population, 72% in this sample); suicidal planning (3.1% in general population, 49% in this sample), and suicide attempts (2.7% in general population, 24% in this sample; [26]. These comparisons remain stark even when examining the subsample who do not have a psychiatric comorbidity, suggesting that this risk is a feature of PMDD itself (see Fig. 1).

**Table 2:** STBs in the full prospectively diagnosed sample and by self-reported psychiatric comorbidity status

STB Lifetime Prevalence Variables	Full PMDD Sample (*N* = 599)		PMDD with Hx of Psychiatric Comorbidity (*N* = 420)		PMDD without Hx of Psychiatric Comorbidity (*N* = 179)		Comparison
	N	%	N	%	N	%	
Ever NSSI	308	51.41	231	55.00	77	43.01	χ2 (1) = 7.21, *p* = .007 Cramer’s V = .11
Ever Passive SI	519	86.64	378	90.00	141	78.77	χ2 (1) = 13.67, *p* < .001 Cramer’s V = .15
Ever Active SI	429	71.61	309	73.57	120	67.03	χ2 (1) = 2.63, *p* = .10 Cramer’s V = .06
Ever Plan an Attempt	291	48.58	217	51.66	74	41.34	χ2 (1) = 5.35, *p* = .02 Cramer’s V = .095
Ever Intend to Attempt	249	41.57	188	44.76	61	34.07	χ2 (1) = 5.89, *p* = .01 Cramer’s V = .10
Ever Prepare for Attempt	239	39.90	179	42.61	60	33.52	χ2 (1) = 4.33, *p* = .03 Cramer’s V = .085
Ever Suicide Attempt	202	33.72	152	36.19	50	27.93	χ2 (1) = 3.82, *p* = .05 Cramer’s V = .08
Ever Hospitalized for STBs	84	14.02	71	16.90	13	7.26	χ2 (1) = 9.67, *p* = .002 Cramer’s V = .13

### Comparing the prevalence of STBs by psychiatric comorbidity status

Table 2 includes statistical comparisons between subsamples, and Fig. 1 includes subgroup bars depicting those with PMDD only and no self-reported psychiatric comorbidities (gray bars), and those with PMDD + at least one self-reported psychiatric comorbidity (red bars). Consistent with our predictions, we observed a significantly greater prevalence of STBs among those with PMDD plus psychiatric comorbidities compared to those with PMDD only, although effect sizes were conventionally small, suggesting that the difference in STB risk between those with and without psychiatric comorbidities may not be clinically significant, and PMDD itself may be characterized by elevated lifetime risk of STBs.

### Influence of demographics and specific psychiatric comorbidities on the presence of lifetime active SI in PMDD

Next, we examined which demographic factors and self-reported lifetime psychiatric diagnoses were most strongly associated with active SI in the full prospectively diagnosed sample. In the first step, predicting lifetime active SI from demographics, parity (i.e., ever having given birth) was associated with lower likelihood of active SI (OR = 0.66, 95% CI: 0.43 to 1). In addition, compared to the modal reference income group ($50,000 to 79,999/yr), individuals were more likely to report active SI if they had income in the $15,000 or lower (USD equivalent) group (OR = 2.08, 95% CI: 1.09 to 3.98) or the $15,999-$19,999 (USD equivalent) group (OR = 2.91, 95% CI: 1.05 to 8.09). In the second step, predicting lifetime active SI from both demographics and psychiatric comorbidities, parity remained significantly associated with lower likelihood of active SI (OR = 0.51, 95% CI: 0.31 to 0.83), and the income range from $30,000 to $34,999 (USD equivalent) emerged as a significant predictor of higher active SI likelihood (OR = 3.03, 95% CI: 1.03 to 8.96). Two comorbid diagnoses significantly predicted higher likelihoods of reporting lifetime active SI: major depressive disorder (OR = 2.06, 95% CI: 1.32 to 3.20) and post-traumatic stress disorder (OR = 2.61, 95% CI: 1.46 to 4.65). Of note, there was also a nonsignificant trend toward lower lifetime active SI among those diagnosed with an anxiety disorder (OR = 0.65, 95% CI: 0.42 to 1.01). There was no evidence of multicollinearity; variance inflation factor values ranged from 1.04 to 1.68). In sum, across models predicting the presence of lifetime active SI among those with prospectively confirmed PMDD, parity was uniquely associated with lower likelihood of active SI, whereas lower income and diagnoses of major depressive disorder and/or post-traumatic stress disorder predicted a higher likelihood of active SI.

### Influence of demographics and specific psychiatric comorbidities on lifetime suicide attempt (among participants reporting lifetime active SI)

Finally, to examine the demographic and psychiatric comorbidity factors that might predict the presence of lifetime suicide attempt among those with prospectively diagnosed PMDD and lifetime active SI (*N* = 429, 72% of the sample), we fit a logistic regression predicting lifetime suicide attempt from demographic and psychiatric comorbidity factors in this subsample. Results are presented in Table 3. In the model predicting likelihood of lifetime attempt from all demographic predictor variables, older age and minoritized sexual orientation were associated with increased risk of attempt among those with lifetime active SI, whereas higher income brackets were associated with reduced risk of attempt; these results were significant controlling for all other demographic predictors shown in Table 3. In the model adding psychiatric comorbidities to the demographic predictor variables, older age remained a significant risk factor, and higher income remained protective. Income between $15,000-$15,999 became a significant risk factor. However, minoritized sexual orientation was no longer a significant risk factor after controlling for psychiatric comorbidities, which may be consistent with the idea that increased risk of suicide attempt in this group is mediated by their heightened risk of psychiatric symptoms/diagnosis. After controlling for demographics, specific psychiatric comorbidities predicting greater risk of attempt among those with SI included PTSD, which was associated with a little over 2 times greater odds of suicide attempt, and borderline PD, which was associated with nearly 5 times greater odds of attempt. There was no evidence of multicollinearity; variance inflation factor values ranged from 1.05 to 1.90).

**Table 3:** Predictors of lifetime suicide attempt among those reporting lifetime active SI in PMDD: logistic regression predicting lifetime suicide attempt from various demographic factors (Model 1) and various demographic factors plus psychiatric comorbidities (Model 2) in the subsample of participants reporting lifetime active suicidal ideation (*N* = 429)

	**Model 1: Demographics**									**Model 2: Psychiatric Comorbidities**										
					Wald Test			95% Confidence interval (odds ratio scale)							Wald Test					95% Confidence interval (odds ratio scale)
	Estimate	SE	OR	z	Wald Statistic	df	p	Lower bound	Upper bound	Estimate	SE	OR		z	Wald Statistic	df	p		Lower bound	Upper bound
(Intercept)	0.336	0.258	1.399	1.299	1.687	1	0.194	0.843	2.321	0.055	0.284	1.056		0.193	0.037	1	0.847		0.605	1.844
Age	**0.279**	**0.127**	**1.322**	**2.196**	**4.822**	**1**	**0.028**	**1.03**	**1.695**	**0.263**	**0.135**	**1.301**		**1.949**	**3.797**	**1**	**0.051**		**0.998**	**1.694**
Gender Minority	0.305	0.686	1.357	0.445	0.198	1	0.656	0.354	5.209	0.294	0.709	1.341		0.414	0.172	1	0.679		0.334	5.383
Racial Minority (Non-white)	-0.113	0.379	0.893	-0.298	0.089	1	0.766	0.425	1.878	-0.155	0.396	0.857		-0.39	0.152	1	0.696		0.394	1.861
Hispanic/Latinx	0.645	0.6	1.905	1.074	1.154	1	0.283	0.588	6.176	0.901	0.634	2.461		1.421	2.02	1	0.155		0.711	8.521
Sexual Orientation Minority	**0.509**	**0.24**	**1.664**	**2.123**	**4.506**	**1**	**0.034**	**1.04**	**2.662**	0.255	0.26	1.29		0.98	0.96	1	0.327		0.775	2.146
Parity	-0.312	0.229	0.732	-1.365	1.863	1	0.172	0.468	1.146	-0.404	0.29	0.668		-1.393	1.941	**1**	0.164		0.378	1.179
income (less than $15,000)	-0.193	0.325	0.824	-0.594	0.353	1	0.552	0.435	1.56	-0.38	0.341	0.684		-1.113	1.239	1	0.266		0.35	1.335
income (Student)	-0.96	0.898	0.383	-1.068	1.141	1	0.286	0.066	2.229	-0.991	0.925	0.371		-1.071	1.147	1	0.284		0.061	2.276
income ($15,000—$19,999)	-0.878	0.455	0.416	-1.928	3.717	1	0.054	0.17	1.015	**-1**	**0.479**	**0.368**		**-2.087**	**4.356**	**1**	**0.037**		0.144	0.941
income ($20,000—$24,999)	0.084	0.485	1.088	0.174	0.03	1	0.862	0.42	2.816	0.154	0.502	1.166		0.307	0.094	1	0.759		0.436	3.117
income ($25,000—$29,999)	-0.2	0.505	0.819	-0.396	0.157	1	0.692	0.304	2.204	-0.18	0.527	0.835		-0.341	0.117	1	0.733		0.297	2.348
income ($30,000—$34,999)	-0.431	0.472	0.65	-0.913	0.833	1	0.361	0.257	1.64	-0.307	0.491	0.736		-0.625	0.391	**1**	0.532		0.281	1.925
income ($35,000—$39,999)	-0.679	0.495	0.507	-1.371	1.878	1	0.171	0.192	1.339	-0.772	0.523	0.462		-1.477	2.182	1	0.14		0.166	1.287
income ($40,000—$49,999)	**-0.841**	**0.366**	**0.431**	**-2.294**	**5.261**	**1**	**0.022**	**0.21**	**0.885**	**-1.007**	**0.388**	**0.365**		**-2.596**	**6.741**	**1**	**0.009**		**0.171**	**0.781**
income ($80,000—$99,999)	**-1.014**	**0.432**	**0.363**	**-2.349**	**5.517**	**1**	**0.019**	**0.156**	**0.845**	**-1.004**	**0.448**	**0.367**		**-2.242**	**5.026**	**1**	**0.025**		**0.152**	**0.881**
income ($100,000 USD +)	**-1.173**	**0.367**	**0.309**	**-3.2**	**10.242**	**1**	**0.001**	**0.151**	**0.635**	**-1.256**	**0.381**	**0.285**		**-3.293**	**10.845**	**1**	** < .001**		**0.135**	**0.601**
Major Depressive Disorder										0.148	0.243	1.16		0.611	0.373	**1**	0.541		0.721	1.866
Peripartum-Onset Depression										-0.109	0.315	0.897		-0.344	0.119	1	0.731		0.484	1.665
Bipolar Disorder										0.646	0.563	1.908		1.147	1.315	1	0.251		0.633	5.753
Eating Disorder										0.272	0.272	1.313		1.001	1.002	1	0.317		0.77	2.237
Borderline Personality Disorder										**1.56**	**0.598**	**4.758**		**2.609**	**6.806**	**1**	**0.009**		1.474	15.361
Attention-Deficit Hyperactivity Disorder										-0.374	0.366	0.688		-1.02	1.041	1	0.308		0.336	1.411
Autism Spectrum Disorder										-0.189	0.777	0.828		-0.243	0.059	1	0.808		0.181	3.796
Anxiety Disorder										0.138	0.248	1.148		0.557	0.31	1	0.578		0.706	1.869
Substance Use Disorder										-0.004	0.342	0.996		-0.012	.00004	1	0.99		0.51	1.945
Post-Traumatic Stress Disorder										**0.752**	**0.262**	**2.122**		**2.871**	**8.241**	**1**	**0.004**		1.27	3.547

## Discussion

### How does lifetime prevalence of STBs in PMDD compare to that of the general population?

We observed high lifetime prevalence rates for STBs in this sample of patients reporting a prospective diagnosis of PMDD. As predicted, lifetime prevalence of active SI (72%) and suicide attempt (34%) were high, and far outpaced lifetime prevalence estimates in cross-national mixed-sex samples of the general population (SI: 9.2%; attempt: 2.7%; *N* = 84,850) [26] and college students (SI: 32.7%; attempt: 4.3%; *N* = 13,984) [27]. They were also higher than an AFAB-specific estimate in another college sample (attempt: 4.6%; *N* = 1,099) [28]. Overall, findings support the hypothesis that PMDD is associated with elevated risk of STBs relative to the general population.

These results have significant implications for training, research, assessment, and treatment. First, training programs in psychiatry, clinical psychology, and gynecology should require basic competency in PMDD assessment and treatment. Currently, mental health providers who are trained to manage suicide risk (e.g., psychiatrists, psychologists) are not systematically trained in detection and treatment of PMDD [29–31], despite evidence that it is a behavioral disorder with a neurobiological etiology [15]. While some training guidelines in gynecology [32] do include requirements to learn basic detection and treatment for the condition, they generally do not receive training in full evidence-based care for PMDD [33], and are not systematically trained in managing patients experiencing STBs [34]. As a result, patients seeking treatment for PMDD may have to choose between a provider who is somewhat knowledgeable about PMDD (gynecology) and a provider who can help them to manage their STBs (psychiatry, psychology). In the future, each of these specialties should be trained in detection, treatment, and collaborative care models for reducing PMDD symptoms and suicide risk alike.

Second, in service of diagnostic clarity, STBs should be considered for inclusion as a diagnostic criterion in the next official DSM diagnostic criteria for PMDD. The current absence of a STB-related diagnostic criterion likely leads to misdiagnosis of PMDD with other disorders that do include STBs in the diagnostic criteria (e.g., borderline PD, MDD). In other words, individuals with true PMDD may be misdiagnosed as having MDD due to the presence of STBs. These misdiagnoses may lead to the use of treatments that do not address the cyclical hormone sensitivities that cause symptoms (including cyclical STBs) in PMDD. Moreover, researchers may continue to exclude PMDD patients with STBs due to concern that STBs signal the presence of a comorbid or alternative disorder, thereby leading to specific types of hormone sensitivity being systematically over or under-included in pathophysiology and treatment studies (e.g., underrepresentation of those with primarily depressive PMDD symptom presentations). Finally, even when PMDD is accurately diagnosed, in the absence of STBs as a diagnostic criterion, clinicians may not appropriately screen PMDD patients for STBs. To circumvent these problems, pending replication of these findings, the relevant DSM working group should consider including STBs as a diagnostic criterion for PMDD.

### Are reports of a lifetime comorbid psychiatric diagnosis associated with lifetime prevalence of STBs in patients with PMDD?

Study findings provide numerous preliminary insights about the impact of psychiatric comorbidity in PMDD. Most notably, most of the sample (70%) reported a lifetime history of diagnosis with a psychiatric comorbidity—even after excluding diagnoses that participants identified as a probable misdiagnosis of PMDD symptoms—suggesting that psychiatric comorbidity was the rule among these (prospectively confirmed) PMDD patients, not the exception. This stands in contrast to typical research practices in the PMDD literature, where those with comorbidities are excluded, and suggests reduced generalizability of existing findings to the general population of patients with PMDD, who are more likely than not to experience a comorbidity. To further elucidate the prevalence and impact of comorbidities in PMDD, there is a need for observational and experimental studies that combine standardized assessment for lifetime comorbidities (e.g., using structured clinical interviews) with prospective assessment and diagnosis of cyclical symptoms.

Although the presence of psychiatric comorbidity was associated with greater risk of STBs in general, Cramer’s V effect sizes indicated negligible-to-weak associations (0.06 to 0.15). This suggests that while psychiatric comorbidities may increase risk of STBs in PMDD, PMDD itself may be associated with a high risk of STBs. This again suggests a risk of misdiagnosis with other psychiatric disorders in which STBs are explicit diagnostic criteria.

Because the presence of comorbidity in PMDD may interfere with accurate diagnosis and treatment of PMDD and related STB risk, rigorous assessment relying on daily symptom ratings is critical. While the DSM-5 does not forbid the presence of a comorbid psychiatric disorder so long as cycling symptoms are different from those of the comorbid disorder [19], Hartlage & Gehlert [22] noted that comorbidity does lead to a “methods dilemma” in which it becomes difficult to accurately diagnose the cycling symptoms of PMDD against the “noise” of background symptoms; as a solution, the authors have described methods for scoring daily symptom ratings that enable the detection of cyclical PMDD symptoms in the context of chronic disorders [16, 22]. Unfortunately, most providers in clinical practice do not use daily symptom ratings to diagnose PMDD [35]- use of daily ratings among providers could bolster clinician confidence in the PMDD diagnosis when comorbidities are present, thereby increasing access to diagnosis and treatment. This is particularly important given that patients with minoritized identities (including race, gender, and sexual orientation) were more likely to report psychiatric comorbidities in our sample, which suggests that they may face more difficulty in obtaining an accurate diagnosis of PMDD.

### Do demographic factors predict the likelihood of suicidal ideation or attempt in prospectively diagnosed PMDD?

In two different models, we examined the role of demographic factors in predicting (1) the lifetime risk of active SI, and (2) the lifetime risk of suicide attempt (among those reporting lifetime active SI only). Lower income was associated with greater risk of lifetime active SI and greater risk of suicide attempt. These findings are consistent with evidence that stressors associated with low SES exacerbate mood disturbances and increase suicide risk [36, 37]. Additionally, although we found that participants with at least one biological child were *more* likely to report a psychiatric comorbidity, we also found that these participants were *less* likely to report lifetime active SI, and less likely to report a suicide attempt. It is unclear whether this represents a protective factor, as we do not have data describing the temporal relationship between the active SI, suicide attempt, and childbirth. In instances where childbirth precedes STBs, a protective effect may arise from having children insofar as children are often cited as reasons for living, or alternatively may represent underreporting of STBs due to fear of being deemed an unfit parent. Advanced age was also associated with higher risk of suicide attempt among those with lifetime active SI, which may represent greater opportunity over more years of illness, or a historic lack of access to treatments. Finally, minoritized sexual orientation was a significant predictor of suicide attempt among those with SI, consistent with minority stress models [25]; however, this effect was likely driven by increased psychiatric symptoms, since it was no longer significant after accounting for comorbidities.

### How do specific self-reported psychiatric comorbidities predict risk of suicidal ideation or attempt in those with PMDD?

Lifetime diagnosis of major depressive disorder or borderline personality disorder were associated with increased reports of lifetime active SI, and both borderline PD and PTSD were associated with risk of suicide attempt among those with lifetime active SI. While these comorbid diagnoses may be accurate, and we did omit diagnoses that participants identified as misdiagnoses, our methods do not allow us to fully rule out the possibility that these comorbidities represented misdiagnosis of severe PMDD with one of these disorders in which STBs are included as an explicit criterion. However, if these comorbidities are genuine, they may indicate a risky developmental trajectory in which monthly PMDD symptoms increase risk for developing chronic syndromes such as MDD, PTSD, and borderline PD. In sum, risk for STBs may be particularly high among patients who experience both cyclical emotional symptoms and chronic symptoms of borderline PD, MDD, or PTSD [22],

Both comorbid borderline personality disorder and PTSD were significant predictors of suicide attempt among those reporting SI. Both disorders are characterized by pervasive, persistent emotion dysregulation and have been suggested to share etiology (e.g., exposure to traumatic events), psychopathology (e.g., emotion-related cognitive bias), and pathophysiology (e.g., arousal dysregulation) [38]. Given that traumatic experiences represent a common theme among these two disorders, it may be that extreme stress exposure increases the risk of suicide attempts among those with SI in the context of PMDD. Indeed, this is consistent with a broader meta-analysis demonstrating that stress exposure predicts suicide attempts among those reporting histories of SI [2]. This also further highlights the possibility that individuals with both PMDD and a history of trauma represent a unique, high-risk group [39, 40].

### Strengths and limitations

This study has numerous strengths. The present sample (*N* = 599) is much larger than most prior studies of PMDD. Additionally, the selection of a subsample reporting a prospective diagnosis of PMDD by a healthcare provider (confirmed using daily ratings) is a significant strength, since prior work has relied on cross-sectional self-reports of PMDD, which are known to lack validity when compared to prospective daily ratings [41, 42]. Additionally, the present study used the Columbia-Suicide Severity Rating Scale [23], a well-validated and comprehensive measure, to characterize the prevalence of various STBs. Finally, the use of an online platform for data collection allowed us to sample from a global population, and the lack of financial compensation for survey completion limited both 1) the likelihood of bot responses and 2) any incentive for participants to misrepresent their experiences or complete the survey without fully reading response options.

There are several significant limitations of this study. First, a diagnosis of PMDD was not confirmed as part of the study protocol, but rather, individuals reported on whether a healthcare provider had used daily symptom ratings to confirm their PMDD diagnosis. Prospective studies in which participants complete daily symptom ratings across two menstrual cycles (to make the PMDD diagnosis as part of the research study) are needed to confirm the findings reported here. Second, comorbid psychiatric diagnoses were self-reported and were not assessed via a structured clinical interview, leaving open the possibility that undiagnosed comorbidities are responsible for some portion of STBs in the present study. Third, our sampling process could have biased the results in this study. While international, our sample predominantly included cisgender, educated, white individuals from developed countries in which English is the primary language (~ 92%). Moreover, while recruitment materials did not mention suicide, recruitment via social media accounts focused on PMDD support and advocacy may have biased the results, since following these PMDD-focused accounts might be correlated with non-PMDD-specific risk factors for suicide (e.g., social isolation, treatment resistance). Future research utilizing online samples should include a clinical control group (e.g., people in an online support group for depression), since our current design cannot parse the unique role of PMDD (vs. engagement in an online support group) in these high rates of STBs. Also, the online sample design involved participants completing surveys in less controlled environments, which may have negatively influenced data quality. Fourth, we asked participants to retrospectively report lifetime psychiatric hospitalization for STBs during the premenstrual phase. While we have no reason to suspect non-truthful reports of hospitalization, our findings relating to premenstrual phase hospitalization must be confirmed with future research using prospective methods.

Finally, our study was cross-sectional, and thus, causality cannot be inferred from study analyses of lifetime variables. The cross-sectional nature of the study also prevents us from disentangling how lifetime STBs and the presence of PMDD diagnoses are temporally related to one another; however, there is no evidence to suggest that people with PMDD do not have cyclical symptoms for their entire reproductive lifespan, and preliminary data suggest that premenstrual symptoms occur as frequently in adolescents as they do in adults. Thus, while we cannot infer causality, we do infer that PMDD symptoms are present for the majority of the individual’s menstrual cycles, and likely overlap with STBs that occurred between menarche and menopause.

## Summary and conclusion

Suicide rates are on the rise among young AFAB individuals in particular [43]. The present study demonstrates a high lifetime prevalence of STBs among AFAB individuals reporting prospectively diagnosed PMDD, and these high rates appear to be present irrespective of psychiatric comorbidities. However, individuals with comorbid PMDD and either MDD or borderline PD appear at greater risk of SI, and those with comorbid PMDD and either borderline PD or PTSD appear to be at greater risk of escalating from active SI to suicide attempt. Demographic factors also played a role in STB risk among those with PMDD, with minoritized sexual orientation, advanced age, nulliparity, and lower income associating with greater risk. If these findings replicate in a study including both prospective daily ratings for PMDD diagnosis and structured evaluation of possible psychiatric diagnoses, they suggest a need for considering STBs as diagnostic criterion for PMDD. There is an urgent need for improved provider training and treatment access for patients with PMDD, and for inclusion of PMDD patients with STBs in pathophysiology and treatment studies.

## Supplementary Information

**Additional file 1: Table 1. **Nonparametric correlations among all study variables.

## Data Availability

The datasets used during the current study, along with code, are available from the corresponding author on reasonable request.
